# A Descriptive Study of Maternal Vaccination Uptake, Attitudes, and Beliefs in Pregnancy Among Persons Delivering at an Urban Safety Net Hospital

**DOI:** 10.1089/whr.2023.0032

**Published:** 2023-06-06

**Authors:** Sheree L. Boulet, Kaitlyn K. Stanhope, Carol DeSantis, Anna Goebel, Julia Dolak, Onyie Eze, Asmita Gathoo, Caroline Braun, Madeline Sutton, Denise J. Jamieson

**Affiliations:** ^1^ Department of Gynecology and Obstetrics, School of Medicine, Emory University, Atlanta, Georgia, USA.; ^2^ Department of Obstetrics and Gynecology, Morehouse School of Medicine, Atlanta, Georgia, USA.

**Keywords:** vaccination, vaccine hesitancy, influenza vaccine, COVID-19 vaccine, pertussis vaccine, whooping cough, pregnancy

## Abstract

**Objective:**

To estimate uptake of influenza, tetanus toxoid, reduced diphtheria toxoid, and acellular pertussis (Tdap), and COVID-19 vaccines during pregnancy and describe vaccine attitudes and beliefs among predominantly racial and ethnic minority individuals delivering at a publicly funded hospital.

**Methods:**

We collected survey and electronic medical record data for English-speaking postpartum individuals who delivered a live-born infant from July 7, 2022, through August 21, 2022, and agreed to participate in our study. The 58-item survey included questions about general vaccine attitudes and beliefs as well as vaccine-specific questions. We calculated rates of influenza, Tdap, and COVID-19 vaccinations and compared distributions of survey responses by number (no vaccines, one vaccine, or two or three of the recommended vaccines) and type of vaccines received during pregnancy.

**Results:**

Of the 231 eligible individuals, 125 (54.1%) agreed to participate. Rates of influenza, Tdap, and COVID-19 vaccination were 18.4%, 48.0%, and 5.6% respectively. A total of 61 (48.8%) did not receive any recommended vaccines during pregnancy, 40 (32.0%) received one vaccine, and 24 (19.0%) received two or three vaccines. Approximately 66.1% of the no vaccine group, 81.6% of the one vaccine group, and 87.5% of the two or three vaccine group strongly agreed or agreed that they trusted the vaccine information provided by their obstetrician or midwife. While most (>69.2%) agreed that the vaccine-preventable diseases were dangerous for pregnant women, only 24.0%, 29.3%, and 40.3% agreed that they were worried about getting influenza, whooping cough, or COVID-19, respectively, while pregnant.

**Discussion:**

Vaccine uptake in our population was low and may be due, in part, to low perceived susceptibility to vaccine-preventable diseases. Obstetricians and midwives were trusted sources of vaccine information, suggesting that enhanced communication strategies could be critical for addressing maternal vaccine hesitancy, particularly in communities of color justifiably affected by medical mistrust.

## Introduction

Maternal vaccination protects expectant mothers and their infants from serious complications caused by infectious diseases. Influenza and tetanus toxoid, reduced diphtheria toxoid, and acellular pertussis (Tdap) vaccines are recommended for all pregnant women in the United States.^1–4^ It is also recommended that all pregnant persons should be vaccinated against COVID-19, including receiving a booster shot.^5,6^ Despite these recommendations and considerable research, public health efforts, and outreach to promote vaccine uptake, maternal vaccination rates have remained suboptimal over time. Rates of seasonal influenza and Tdap vaccination have stayed at ∼50% or less for at least a decade,^7–10^ and over one quarter of pregnant persons are still not fully vaccinated against COVID-19.^11^

There are also persistent racial and ethnic disparities in maternal vaccine acceptance. In 2020–2021, 41% of black pregnant persons were vaccinated against influenza, compared with 54% of white pregnant persons.^7^ Similarly, Tdap vaccination rates lag among black and Hispanic pregnant persons (27% and 48%, respectively) compared with their white counterparts (60%).^7^ Available data also suggest that black pregnant persons have the lowest coverage of the COVID-19 vaccine series, although overall uptake is higher than for influenza or Tdap.^11^

Decisions regarding vaccination are complex and are influenced by perceptions of vaccine effectiveness and safety, social and contextual factors, and individual knowledge, beliefs, and experiences.^12,13^ For communities of color, vaccine confidence is tempered by justified mistrust of medicine and science due to a long history of unethical research and practices in vulnerable populations as well as experiences of racism in their daily lives.^14–16^ Understanding the drivers of maternal vaccine decision-making is critical to developing effective clinical and population health interventions for addressing vaccine hesitancy. However, most studies of maternal vaccine attitudes and beliefs to date, including those focused on characterizing racial and ethnic disparities, included mostly white pregnant persons.^13,17–20^

Results from one recent study suggest that less than one-third of black survey respondents believed that flu and Tdap shots were important for the health and safety of themselves and their baby, compared with more than 60% of white respondents.^21^ The goal of this study was to describe maternal vaccine attitudes and beliefs and uptake of influenza, Tdap, and COVID-19 vaccines among predominantly racial and ethnic minority postpartum individuals who delivered a live-born infant between July 7, 2022, and August 21, 2022.

## Materials and Methods

Using data from daily delivery logs and electronic medical records (EMR), we identified all live-birth deliveries to English-speaking pregnant persons 18 years of age or older occurring during a 6-week period (July 7, 2022, through August 21, 2022) at Grady Memorial Hospital, a large publicly funded hospital in Atlanta, Georgia. Approximately 68% of individuals delivering at Grady report their race and ethnicity as non-Hispanic black and 23% are non-English speakers.^22^ Individuals who were COVID-19 positive at delivery were not eligible to participate. Eligible participants were approached by study staff during their postpartum stay and invited to complete a survey on vaccine attitudes and beliefs. Individuals who consented to participate completed an electronic survey using a tablet or their personal smartphone.

Surveys took ∼15 minutes to complete and included questions on demographic characteristics (self-reported race and ethnicity, marital status, educational status, household composition, employment during pregnancy, and languages spoken at home) and attitudes and beliefs regarding influenza, Tdap, and COVID-19 vaccines.

Measures of vaccine attitudes and beliefs were derived from a previously published instrument^23^ and included 58 items that measured descriptive and injunctive norms,^24^ self-efficacy, trust in information sources, and perceived susceptibility and severity of influenza, Tdap, and COVID-19 (Appendix A1). Responses were reported using a Likert-type scale (strongly agree, agree, neutral, disagree, strongly disagree). Information on vaccination status and clinical characteristics was extracted from the EMR. The EMR imports information from the Georgia Registry of Immunization Transactions and Services, which should include all vaccines administered in the state of Georgia, regardless of whether they occurred in our health system or another place. Prenatal care utilization was defined using the Kotelchuck index^25^; however, we were unable to quantify utilization for individuals receiving some or all of their care outside of our health system.

We included influenza vaccines given any time during pregnancy, Tdap vaccines given between 27 and 36 weeks of gestation, and 1 or more COVID-19 vaccines given any time before or during pregnancy. Vaccinations given at or after the delivery were not included in the rate calculations.

We calculated vaccination rates for each of the vaccines and compared the distribution of demographic and clinical characteristics for individuals who received no vaccines, one vaccine, or two or three of the recommended vaccines during their pregnancy. For general questions on vaccine attitudes and beliefs, we described the distribution of all survey responses (strongly agree, agree, neutral, disagree, strongly disagree). For question about specific vaccines or associated diseases, we described the proportion of respondents who indicated that they strongly agreed or agreed with the survey statements. We used Fisher's exact tests to generate *p*-values.

The study was approved by Emory's Institutional Review Board (no. 00004569) and the Grady Research Oversight Committee. SAS version 9.4 was used for all analyses.

## Results

There were 308 deliveries between July 7, 2022, and August 21, 2022. Of those, 77 were determined to be ineligible for the study (52 did not speak English and 25 were COVID-19 positive), and 54.1% (125/231) individuals agreed to participate. Among the 308 individuals who delivered during the study period, the rates of influenza, Tdap, and COVID-19 vaccination were 18.2%, 41.9%, and 5.5%, respectively. Among the 125 individuals who consented to participate in the study, the rates of influenza, Tdap, and COVID-19 vaccination were 18.4%, 48.0%, and 5.6% respectively. All of the seven participants who received the COVID-19 vaccine had at least one dose during pregnancy. Approximately 48.8% (*n* = 61) did not receive any of the recommended vaccines during pregnancy, 32.0% (*n* = 40) received one vaccine, and 19.0% (*n* = 24) received two or three vaccines.

Of the three vaccines recommended during pregnancy, uptake of Tdap was highest for individuals receiving one (92.5%) and two or three vaccines (95.8%) (Table 1).

**Table 1.** Demographic and Clinical Characteristics of the Study Population by Receipt of Influenza, Tetanus Toxoid, Reduced Diphtheria Toxoid, and Acellular Pertussis, and COVID-19 Vaccines During Pregnancy

Characteristic	No vaccines ***n*** = 61	One vaccine ***n*** = 40	Two or three vaccines ***n*** = 24	***p***
	***n*** (%)	***n*** (%)	***n*** (%)	
Vaccine type				
Influenza	—	3 (7.5)	20 (83.3)	<0.0001
Tdap	—	37 (92.5)	23 (95.8)	1.00
COVID-19	—	0	7 (29.2)	0.0006
Race/ethnicity				0.20
Hispanic	5 (8.6)	2 (5.1)	5 (20.8)	
Non-Hispanic black	48 (82.8)	35 (89.7)	16 (66.7)	
Non-Hispanic white	2 (3.4)	2 (5.1)	1 (4.2)	
Non-Hispanic other and multiracial	3 (5.2)	0	2 (8.3)	
Missing/unknown	3	1	0	
Age (years)				0.84
<20	8 (13.1)	7 (17.5)	2 (8.3)	
20–24	15 (24.6)	6 (15.0)	5 (20.8)	
25–29	18 (29.5)	14 (35.0)	6 (25.0)	
30–34	14 (23.0)	9 (22.5)	6 (25.0)	
35+	6 (9.8)	4 (10.0)	5 (20.8)	
Age (mean, SD)	26.6 (6.0)	26.7 (5.8)	28.5 (6.5)	
Health insurance				0.62
Private	6 (9.8)	5 (12.5)	4 (16.7)	
Public	55 (90.2)	35 (87.5)	20 (83.3)	
Marital status				0.77
Married/cohabitating	29 (54.7)	17 (47.2)	15 (65.2)	
Divorced/separated	3 (5.7)	3 (8.3)	1 (4.3)	
Never married	21 (39.6)	16 (44.4)	7 (30.4)	
Missing	8	4	1	
Lived in same house while pregnant				
Husband/partner	30 (50.8)	19 (48.7)	18 (78.3)	0.05
Children <6 years	16 (27.1)	12 (30.8)	7 (30.4)	0.90
Children ≥6 years	20 (33.9)	11 (28.2)	4 (17.4)	0.33
Other	25 (42.4)	15 (38.5)	6 (26.1)	0.41
Lived alone	5 (8.5)	3 (7.7)	1 (4.3)	1.00
Missing	2	1	1	
Education				0.31
<High school	9 (15.2)	4 (10.3)	3 (13.0)	
High school	35 (59.3)	17 (43.6)	11 (47.8)	
Some college or college graduate	15 (25.4)	18 (46.1)	9 (39.1)	
Language spoken at home				0.01
Only English	57 (93.4)	37 (92.5)	17 (70.8)	
English and another	3 (4.9)	1 (2.5)	6 (25.0)	
Another only	1 (1.6)	2 (5.0)	1 (4.2)	
Worked for pay during pregnancy				0.94
Full-time	23 (41.1)	17 (43.6)	12 (50.0)	
Part-time	14 (25.0)	9 (23.1)	6 (25.0)	
None	19 (33.9)	13 (33.3)	6 (25.0)	
Missing	5	1	0	
Parity				0.81
0	21 (34.4)	15 (37.5)	9 (37.5)	
1	20 (32.8)	10 (25.0)	9 (37.5)	
2+	20 (32.8)	15 (37.5)	6 (25.0)	
Comorbid conditions				
Hypertension	5 (8.2)	7 (17.5)	3 (12.5)	0.36
Asthma	11 (18.0)	8 (22.5)	8 (33.3)	0.29
Obesity	5 (8.2)	14 (35.0)	10 (41.7)	0.0002
Mental health disorder	3 (4.9)	9 (22.5)	4 (16.7)	0.02
Substance use disorder	4 (6.6)	5 (12.5)	1 (4.2)	0.51
Tobacco use	3 (5.2)	4 (10.0)	2 (8.3)	0.66
Anemia	15 (24.6)	10 (25.0)	5 (20.8)	0.92
Prenatal care utilization				0.001
≥Adequate	11 (18.0)	15 (37.5)	18 (75.0)	
Intermediate	6 (9.8)	7 (17.5)	5 (20.8)	
Inadequate	17 (27.9)	17 (42.5)	1 (4.2)	
Unknown^a^	27 (44.3)	1 (2.5)	1 (4.2)	
Delivery provider type				0.88
Resident	29 (47.5)	20 (50.0)	14 (58.3)	
Attending	31 (50.8)	20 (50.0)	10 (41.7)	
CNM	1 (1.6)	0	0	
COVID-19 infection during pregnancy	1 (1.6)	2 (5.0)	1 (4.2)	0.52

^a^ No documented prenatal care at Grady.

CNM, certified nurse midwife; SD, standard deviation; Tdap, tetanus toxoid, reduced diphtheria toxoid, and acellular pertussis.

Individuals receiving no vaccines were more likely than those receiving two or three vaccines to speak only English (93.4% vs. 70.8%) and less likely to live with their husband or partner (50.8% vs. 79.3%) (Table 1). The prevalence of comorbid conditions including obesity and mental health disorders was also lower among individuals receiving no vaccines compared with those receiving any vaccines.

Nearly two-thirds (66.1%) of the no vaccine group, 81.6% of the one vaccine group, and 87.5% of the two or more vaccine group strongly agreed or agreed that they trusted the vaccine information provided by their obstetrician or midwife (Table 2). Overall, reported trust in other sources of vaccine information (*e.g.,* scientists and doctors at universities and federal agencies) was lower than trust in an individual's obstetric care provider. Nearly all respondents (>95%) agreed that they had control over whether they received vaccines during their pregnancy. Notably, 73.9% of individuals receiving two or three vaccines agreed that the majority of their friends or family would encourage them to get vaccinated during pregnancy compared with 47.5% in the one vaccine group and 33.3% in the no vaccine group.

**Table 2.** General Vaccine Attitudes and Beliefs

Statement	No vaccines ***n*** = 61	One vaccine ***n*** = 40	Two or three vaccines ***n*** = 24	***p***
I trust the information provided by my obstetrician or midwife about vaccines during pregnancy.				0.02
Strongly agree/agree	39 (66.1)	31 (81.6)	21 (87.5)	
Neutral	14 (23.7)	7 (18.4)	3 (12.5)	
Strongly disagree/disagree	6 (10.2)	0	0	
Missing	2	2	0	
I trust the information provided by federal agencies such as the CDC about vaccines during pregnancy.				0.36
Strongly agree/agree	33 (55.0)	25 (67.6)	14 (60.9)	
Neutral	17 (28.3)	10 (27.0)	9 (39.1)	
Strongly disagree/disagree	10 (15.7)	2 (5.4)	0	
Missing	1	3	1	
I trust the information provided by scientists and doctors at universities and academic institutions about vaccines during pregnancy.				0.16
Strongly agree/agree	22 (53.4)	23 (62.2)	17 (70.8)	
Neutral	18 (31.0)	13 (35.1)	7 (29.2)	
Strongly disagree/disagree	9 (15.5)	1 (2.7)	0	
Missing	3	3	0	
It is in my control whether or not I get vaccines during my pregnancy.				0.87
Strongly agree/agree	57 (95.0)	39 (100)	24 (100)	
Neutral	2 (3.3)	0	0	
Strongly disagree/disagree	1 (1.7)	0	0	
Missing	1	1	0	
The majority of my friends and family would get the vaccines that are recommended during pregnancy.				0.02
Strongly agree/agree	22 (36.7)	18 (47.4)	16 (66.7)	
Neutral	20 (33.3)	14 (36.8)	7 (29.2)	
Strongly disagree/disagree	18 (30.0)	6 (15.8)	1 (4.2)	
Missing	1	2	0	
The majority of my friends and family would encourage me to get the vaccines that are recommended during pregnancy.				0.14
Strongly agree/agree	20 (33.3)	19 (47.5)	17 (73.9)	
Neutral	23 (38.3)	15 (37.5)	3 (13.0)	
Strongly disagree/disagree	17 (28.3)	6 (15.0)	2 (13.0)	
Missing	1	0	1	
I have most of the important information I need to make a decision about vaccines given during pregnancy.				0.43
Strongly agree/agree	46 (76.7)	33 (89.8)	21 (91.3)	
Neutral	10 (16.7)	3 (7.9)	2 (8.7)	
Strongly disagree/disagree	4 (6.7)	2 (5.3)	0	
Missing	1	2	1	

CDC, Centers for Disease Control and Prevention.

**FIG. 1.**
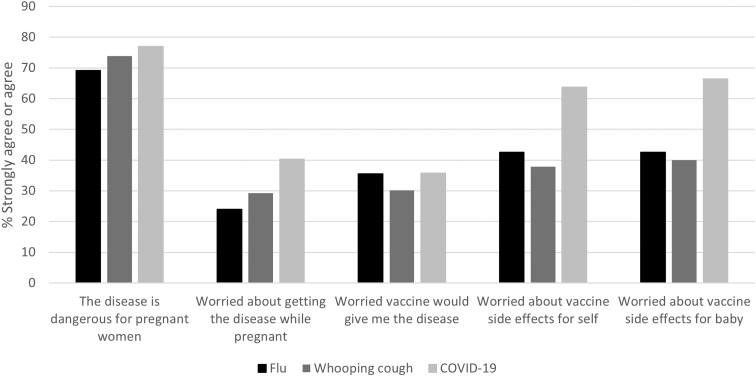
Perceptions of influenza, whooping cough, and COVID-19 severity, susceptibility, and vaccination barriers. This graph depicts the percentage of respondents who agreed or strongly agreed with statements regarding influenza, whooping cough, and COVID-19.

Most individuals agreed that influenza, whooping cough, and COVID-19 are dangerous for pregnant women (Fig. 1). However, only 24.0%, 29.3%, and 40.3% were worried about getting influenza, whooping cough, and COVID-19, respectively, during their pregnancy. Nearly two-thirds of respondents reported concerns about COVID-19 vaccine side effects for themselves and their babies.

**FIG. 2.**
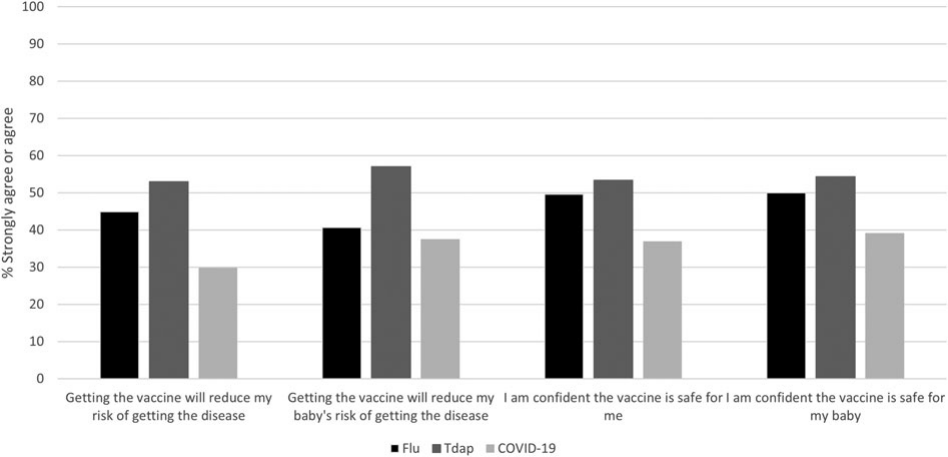
Perceptions of influenza, Tdap, and COVID-19 vaccine effectiveness and safety. This graph depicts the percentage of respondents who agreed or strongly agreed with statements about the effectiveness and safety of influenza, Tdap, and COVID-19 vaccines. Tdap, tetanus toxoid, reduced diphtheria toxoid, and acellular pertussis.

Perceived vaccine efficacy was highest for the Tdap vaccine, with more than half of the individuals agreeing that vaccination would reduce risk of getting whooping cough for themselves or their babies (Fig. 2). Only 29.8% agreed that getting the COVID-19 vaccine would reduce their risk of getting COVID-19 and 37.5% agreed that it would reduce their baby's risk. Roughly half of all respondents agreed that the influenza and Tdap vaccines were safe for themselves and their babies.

## Discussion

Among postpartum individuals delivering at a publicly funded hospital between July 7, 2022, and August 21, 2022, we documented remarkably low rates of influenza, Tdap, and COVID-19 vaccine uptake relative to national rates. In addition, we found that individuals refusing all recommended vaccines during pregnancy tended to be younger, more often publicly insured, identify as non-Hispanic black, and had lower prevalence of chronic conditions than individuals receiving one or more vaccines. Most respondents perceived vaccine information provided by their obstetrician or midwife to be trustworthy; however, there was evidence that support from family and friends may play an important role in shaping vaccine decisions during pregnancy.

While most individuals agreed that influenza, whooping cough, and COVID-19 were dangerous for pregnant women, perceived risk of getting the diseases was low and there was considerable concern about side effects associated with the COVID-19 vaccine. Similarly, the COVID-19 vaccine was perceived to be least effective and the least safe among the three vaccines recommended for pregnant people.

Rates of maternal influenza, Tdap, and COVID-19 vaccination in this study (18.1%, 41.9%, and 5.5%, respectively) were much lower than the most recent national estimates (54.5%,^7^ 53.5%,^7^ and 71.2%,^11^ respectively). Furthermore, influenza and Tdap vaccine uptake was markedly lower than that reported in prior studies for deliveries taking place at the same institution between 2016 and 2018 (50%^22^ and 65%,^26^ respectively), suggesting a dramatic decrease in maternal vaccine acceptance in recent years. The reason for this decline is not known; however, similar patterns have been noted in national data and may be due to increased safety concerns, mounting vaccine mistrust, and disruptions in access to preventive care in the context of the COVID-19 pandemic.^27^

Consistent with current evidence,^13,28–30^ we found that obstetric care providers were the most trusted source of vaccine information for our pregnant patients. The apparent disconnect between relatively high reported rates of provider trust and low vaccine uptake suggests that other factors may play a role in influencing vaccine decision-making in our population. Social norms may be an important driver as more than 28% of individuals who were not vaccinated believed that the majority of their friends and family would not encourage vaccination during pregnancy. Findings from other studies suggest that social norms are highly predictive of vaccine acceptance, particularly for black communities where family culture was found to have a strong impact on decisions regarding influenza vaccination.^18,31,32^ It is also likely that social media contributed to individual beliefs regarding vaccine safety and trust during this time period.^33^

Despite endorsing beliefs that influenza, whooping cough, and COVD-19 are dangerous for pregnant women, perceived susceptibility was relatively low in our population and largely overshadowed by concerns about side effects, especially in relation to the COVID-19 vaccine. Overall, the Tdap vaccine was considered the safest and most effective vaccine, a finding that is consistent with other studies.^18,34^ The reasons for higher rates of confidence in the Tdap vaccine are not known but may be because there are fewer prevailing myths and misperceptions surrounding the vaccine as it is only given during pregnancy.

Our findings are subject to several limitations. Because our study was limited to pregnant persons delivering at a single public hospital during a 6-week time period in the summer, our findings may not be generalizable to other pregnant populations and those delivering during other seasons. Our results may be affected by selection bias if individuals who agreed to participate were systematically different than those who refused. Our response rate (54.1%) was lower than the typical threshold of 60% but was higher than those reported for the Centers for Disease Control and Prevention's National Immunization Survey-Flu (21%–25%)^35^ and consistent with other vaccine belief studies among pregnant and postpartum persons.^17,36^

Reassuringly, vaccination rates in our study were similar among those who agreed to participate and those who did not, suggesting that our study population was not markedly different than the general population of persons delivering at our institution. Non-English-speaking participants were ineligible to participate in the study; thus, we were unable to characterize vaccine knowledge and beliefs in a population that faces unique cultural and language barriers to vaccination. In addition, small sample size limited our ability to examine patterns among specific subgroups of study participants. Finally, because of the timing of the survey, some participants may not have been pregnant during flu season and thus were not eligible to receive an influenza vaccination.

Maternal vaccine uptake in this population of predominantly non-Hispanic black pregnant persons delivering at an urban safety net hospital was remarkably low relative to the national rates and prior studies in the same population,^7,11,22,26^ suggesting a potential shift in attitudes in the context of the COVID-19 pandemic. Although obstetricians and midwives were the most trusted source of vaccine information, low perceived susceptibility and efficacy in addition to safety concerns highlight a critical need for more effective communication strategies as part of routine prenatal care. For racial and ethnic minority populations in particular, the provider–patient relationship can engender both trust and distrust, and there is growing evidence that acknowledging power dynamics and conveying empathy are key to overcoming vaccine hesitancy.^37,38^

Other strategies for improving maternal vaccine uptake include highlighting the benefits of maternal vaccination for infants,^39^ use of motivational interviewing,^40^ and revisiting vaccination at subsequent prenatal visits for patients who refuse initially as vaccine hesitancy may change over the course of pregnancy. Leveraging social media influencers and promoting social norms related to the benefits of maternal vaccination are also promising strategies for increasing acceptance.^32,41^ Our finding that more respondents were concerned about getting whooping cough than influenza during pregnancy suggests that there is room for additional patient education regarding risks and transmission of vaccine-preventable diseases.

Other studies have found that pregnant persons preferred to wait until after the second trimester of pregnancy or breastfeeding to receive the COVID-19 vaccine, suggesting a need for better education regarding the protective effect of vaccines for both mothers and babies.^42^ Lastly, addressing racism and discrimination in the health care setting is critically important, as perceived inequities in treatment contribute to distrust and discourage vaccination.^43^

## Data Statement

Due to the sensitive nature of the questions asked in this study, survey respondents were assured that raw data would remain confidential and would not be shared.

## Abbreviations Used

CDC: Centers for Disease Control and Prevention
CNM: certified nurse midwife
EMR: electronic medical records
SD: standard deviation
Tdap: tetanus toxoid, reduced diphtheria toxoid, and acellular pertussis

## Appendix A1. Survey Questions

### Section 1. Respondent Characteristics

1. Which one or more of these groups would you say is your race? *Check all that apply*
  a. Black or African American
  b. White
  c. Asian
  d. American Indian/Alaska Native
  e. Native Hawaiian/Pacific Islander
  f. Other
2. Are you of Hispanic, Latina, or Spanish origin?
  a. Yes
  b. No
3. Are you currently:
  a. Married
  b. Not married but living with my partner
  c. Divorced
  d. Widowed
  e. Separated
  f. Never married
4. What is the highest grade or year of school you completed?
  a. Never attended school
  b. Grades 1 through 8
  c. Grades 9 through 11
  d. Grade 12 (high school graduate)
  e. Some college or technical school (1 to 3 years)
  f. College 4 years or more (college graduate)
5. Who lived in the same house with you while you were pregnant? Check ALL that apply
  a. My husband or partner
  b. Children aged younger than 12 months: How many children?
  c. Children aged 1 year to 5 years: How many children?
  d. Children aged 6 years and older: How many children?
  e. My mother
  f. My father
  g. My husband's or partner's parent(s)
  h. Friend or roommate
  i. Other family members or relatives
  j. Other: Please tell us:
  k. I lived alone
6. At any time during your pregnancy, did you work at a job for pay?
  a. Yes, I worked full-time (30 or more hours/week)
  b. Yes, I worked part-time (less than 30 hours per week)
  c. No
7. What language(s) do you usually speak at home?
  a. Only English
  b. English and another language, please tell us what other language:
  c. Only another language, please tell us what other language:

### Section 2. Vaccine Attitudes and Beliefs

We are interested in how you felt about the following statements during your pregnancy. Please read each statement carefully and indicate how you feel about each statement.

**Table:**

	Strongly agree	Agree	Neutral	Disagree	Strongly disagree
I am confident that getting the flu vaccine during my pregnancy is safe for me.					
I am confident that getting the flu vaccine during my pregnancy is safe for my unborn baby.					
I am confident that getting the whooping cough vaccine during my pregnancy is safe for me.					
I am confident that getting the whooping cough vaccine during my pregnancy is safe for my unborn baby.					
I am confident that getting the COVID-19 vaccine during my pregnancy is safe for me.					
I am confident that getting the COVID-19 vaccine during my pregnancy is safe for my unborn baby.					
I trust the information provided by my obstetrician or midwife about vaccines during pregnancy.					
I trust the information provided by federal agencies such as the CDC about vaccines during pregnancy.					
I trust the information provided by scientists and doctors at universities and academic institutions about vaccines during pregnancy.					
I worried that I would get the flu while I was pregnant.					
The flu is dangerous for pregnant women.					
The flu is more dangerous for pregnant women than for women who are not pregnant.					
Getting the flu vaccine will reduce my risk of getting the flu during my pregnancy.					
Getting the flu vaccine while I am pregnant will reduce my unborn baby's risk of getting the flu.					
I worried about the side effects of the flu vaccine for myself.					
I worried about the side effects of the flu vaccine for my baby.					
I worried that the flu vaccine would give me the flu.					
I worried that I would get whooping cough while I was pregnant.					
Whooping cough is dangerous for pregnant women.					
Whooping cough vaccine will reduce my chances of getting whooping cough.					
Whooping cough vaccine will reduce the chance of me giving whooping cough to my unborn baby.					
Getting the whooping cough vaccine while I am pregnant will reduce my unborn baby's risk of getting whooping cough.					
I worried about the side effects of the whooping cough vaccine for myself.					
I worried about the side effects of the flu whooping cough vaccine for my baby.					
I worried that the whooping cough vaccine would give me whooping cough.					
I worried that I would get COVID-19 while I was pregnant.					
COVID-19 is dangerous for pregnant women.					
COVID-19 vaccine will reduce my chances of getting COVID.					
COVID-19 vaccine will reduce the chance of me giving COVID to my unborn baby.					
Getting the COVID-19 vaccine while I am pregnant will reduce my unborn baby's risk of getting COVID.					
I worried about the side effects of the CVOD-19 vaccine for myself.					
I worried about the side effects of the COVID-19 vaccine for my baby.					
I worried that the COVID-19 vaccine would give me COVID.					
It is in my control whether or not I get vaccines during my pregnancy.					
The majority of my friends and family would get the vaccines that are recommended during pregnancy.					
The majority of my friends and family would encourage me to get the vaccines that are recommended during pregnancy.					
I have most of the important information I need to make a decision about vaccines given during pregnancy.					
I know enough about the safety of the flu vaccine to make a decision about getting the vaccine for myself while pregnant.					
I know enough about the safety of the whooping cough vaccine to make a decision about getting the vaccine for myself while pregnant.					

CDC, Centers for Disease Control and Prevention.
